# Dynamic changes in gene expression through aging in *Drosophila melanogaster* heads

**DOI:** 10.1093/g3journal/jkaf039

**Published:** 2025-02-24

**Authors:** Katherine M Hanson, Stuart J Macdonald

**Affiliations:** Department of Molecular Biosciences and Center for Genomics, University of Kansas, 1200 Sunnyside Avenue, Lawrence, KS 66045, USA; Department of Molecular Biosciences and Center for Genomics, University of Kansas, 1200 Sunnyside Avenue, Lawrence, KS 66045, USA

**Keywords:** gene expression, lifespan, aging, gene ontology enrichment, FlyBase

## Abstract

Work in many systems has shown large-scale changes in gene expression during aging. However, many studies employ just 2 arbitrarily chosen timepoints to measure expression and can only observe an increase or a decrease in expression between “young” and “old” animals, failing to capture any dynamic, nonlinear changes that occur throughout the aging process. We used RNA sequencing to measure expression in male head tissue at 15 timepoints through the lifespan of an inbred *Drosophila melanogaster* strain. We detected >6,000 significant, age-related genes, nearly all of which have been seen in previous *Drosophila* aging expression studies and that include several known to harbor lifespan-altering mutations. We grouped our gene set into 28 clusters via their temporal expression change, observing a diversity of trajectories; some clusters show a linear change over time, while others show more complex, nonlinear patterns. Notably, reanalysis of our dataset comparing the earliest and latest timepoints—mimicking a 2-timepoint design—revealed fewer differentially expressed genes (around 4,500). Additionally, those genes exhibiting complex expression trajectories in our multitimepoint analysis were most impacted in this reanalysis; their identification, and the inferred change in gene expression with age, was often dependent on the timepoints chosen. Informed by our trajectory-based clusters, we executed a series of gene enrichment analyses, identifying enriched functions/pathways in all clusters, including the commonly seen increase in stress- and immune-related gene expression with age. Finally, we developed a pair of accessible Shiny apps to enable exploration of our differential expression and gene enrichment results.

## Introduction

Aging is marked by both a decline in organismal function and an increased risk for disease. As complex traits, both environmental and genetic factors contribute to variation in lifespan and health at old age. Identifying these factors, and their impact on lifespan and healthspan, enables understanding of the negative effects of aging, which is increasingly important as populations throughout the world age. In humans, the estimated heritability of longevity is 7–30% (Mayer 1991; Herskind *et al*. 1996; Kaplanis *et al*. 2018; Ruby *et al*. 2018), and genome-wide association studies (GWASs) have been used to study the genetic basis of aging (Newman *et al*. 2010; Deelen *et al*. 2011, 2014; Sebastiani *et al*. 2012, 2017; Broer *et al*. 2015; Joshi *et al*. 2016, 2017; Pilling *et al*. 2016, 2017; Zeng *et al*. 2016; Timmers *et al*. 2019; Melzer *et al*. 2020). GWAS often rely on examining sets of individuals who are >90 years old (Newman *et al*. 2010; Deelen *et al*. 2011, 2014; Sebastiani *et al*. 2012, 2017; Zeng *et al*. 2016), but this can constrain the sample size and power of such studies (Tan *et al*. 2008; Newman and Murabito 2013). Creative approaches have enabled markedly increased sample size—for instance, Joshi *et al*. (2016) associate offspring genotype with parental lifespan phenotype—yet human lifespan GWAS have only discovered a handful of replicable associations, for instance near APOE, FOXO3, and CHRNA3/5 (Deelen *et al*. 2011; Broer *et al*. 2015; Joshi *et al*. 2016, 2017; Pilling *et al*. 2016; Timmers *et al*. 2019).

Outside of a deficit of power in genetic studies, studying aging directly in humans can be difficult due to uncontrollable environmental variables, many of which can impact lifespan. For example, calorie restriction has been shown to increase lifespan and health in model organisms (McCay *et al*. 1939; Pletcher *et al*. 2002; Cypser *et al*. 2013; Mattison *et al*. 2017), and in humans a 2-year calorie restriction study showed a reduction in cardiometabolic risk factors including cholesterol and blood pressure (Kraus *et al*. 2019). However, effectively studying the impact of diet on lifespan in humans is challenging due to the difficulty accurately measuring calorie intake, the lack of adherence by participants, and several other concerns (Hu 2024). Ideally, studies investigating the genetic and molecular contributions to aging would limit all other sources of variation, something that is not feasible or practical in human studies.

Model organisms have proven useful conduits to study aging due to their shorter lifespans, ease of testing many individuals, and the ability to control both environmental and genetic variation. Both genetic and environmental contributors to aging have been successfully identified in vertebrate model systems (McCay *et al*. 1939; Gocmez *et al*. 2020; Cai *et al*. 2022), in invertebrates such as *Drosophila melanogaster* and *Caenorhabditis elegans* (Pletcher *et al*. 2002; McCarroll *et al*. 2004; Kenyon 2010; Highfill *et al*. 2017), and in the fungi *Saccharomyces cerevisiae* and *Podospora anserina* (Philipp *et al*. 2013; Hu *et al*. 2014). These models have helped identify and understand lifespan-associated genes and systems that have relevance for human populations. For instance, FOXO3—a member of the forkhead box transcription factor O (FOXO) family—has been implicated in human aging via GWAS (Willcox *et al*. 2008; Deelen *et al*. 2014; Broer *et al*. 2015), and studies in *C. elegans* and *D. melanogaster* have also identified and characterized FOXO family genes that impact lifespan (Kimura *et al*. 1997; Lin *et al*. 1997; Giannakou *et al*. 2007; Alic *et al*. 2014; Taormina *et al*. 2019).

Cellular processes do not remain static over an individual's lifetime, and instead change, adapt, and sometimes break down over time. By measuring these changes, we can understand the cellular and physiological phenomena that are affected by aging. A common strategy to assess such age-related changes is with the use of gene expression analyses. These analyses often compare the genome-wide gene expression profile between “young” and “old” individuals, and identify genes that are differentially expressed with age. This approach has been successfully employed in multiple systems (Lu *et al*. 2004; Wilson *et al*. 2015; Bordet *et al*. 2021; Wang *et al*. 2022), and has resulted in expression-based hallmarks of aging (Frenk and Houseley 2018), including increased expression of stress response and immunity genes (Lu *et al*. 2004; Bajgiran *et al*. 2021; Bordet *et al*. 2021; Wang *et al*. 2022), and decreased expression of genes associated with mitochondria and the electron transport chain (ETC) (Lu *et al*. 2004; Bordet *et al*. 2021).

A challenge with 2-timepoint, young vs old comparisons is that there is little consistency in the definitions of “young” and “old”, making comparisons across studies difficult. For instance, various studies in *D. melanogaster* have used “young” flies sampled between 1 and 10 days old, and “old” flies that were 35 to 61 days old (Landis *et al*. 2004; Girardot *et al*. 2006; Carnes *et al*. 2015; Bajgiran *et al*. 2021; Bordet *et al*. 2021). While in some cases the “old” timepoint is determined by the survivorship of the aging cohort, rather than by chronological age (Landis *et al*. 2004; Doroszuk *et al*. 2012; Highfill *et al*. 2017). Additionally, 2-timepoint expression analyses are limited to making binary conclusions about expression change; expression either increases or decreases with age. Aging is a complex process, and this simplification may often fail to yield an accurate picture of age-related expression for most genes. Indeed, work in several systems assaying various molecular phenotypes at multiple timepoints throughout lifespan have identified many genes and gene products with nonlinear trajectories (Lund *et al*. 2002; Pletcher *et al*. 2002; Remondini *et al*. 2010; Gheorghe *et al*. 2014; Haustead *et al*. 2016; Schaum *et al*. 2020; Xie *et al*. 2022; Olecka *et al*. 2024; Shen *et al*. 2024 Aug 14).

Here we set out to robustly measure expression trajectories throughout the adult lifespan of an inbred *D. melanogaster* strain, focusing on head tissue from males. Flies were aged for 59 days, and sampled for RNAseq at 15 timepoints. We recorded the number of deaths in our aging cohort daily, allowing us to calculate a survival-based “physiological age” in addition to the “chronological age” for each time point, and endeavored to sample roughly evenly through both metrics. Subsequently, using multiple analyses we identified genes whose expression changed with aging, and clustered differentially expressed genes based on their expression trajectories. With the idea that genes with similar expression trajectories may share similar functional roles (e.g. Zhang *et al*. 2004), we did a series of enrichment analyses for each of our clusters, identifying numerous enriched pathways. To better understand the value of a multitimepoint expression study, we subsequently reanalyzed our data in a 2-timepoint, young vs old analysis framework. Finally, we compared the results of our work with a series of prior expression-based *D. melanogaster* aging studies.

## Materials and methods

### Fly rearing, maintenance, and aging

We employed a single inbred *D. melanogaster* strain, A4, which is one of the founders of the *Drosophila* Synthetic Population Resource (King *et al*. 2012; Chakraborty *et al*. 2018), a panel of recombinant strains enabling the genetic characterization of complex traits. Following several generations of expansion, we generated 200 replicate vials of the A4 strain, clearing adults to maintain roughly even egg/larval densities over vials. In the following generation we harvested 0–2-day-old A4 males over CO_2_ anesthesia, collecting 132 vials of 40 males (for a subsequent collection of aged animals for RNAseq) and 5 vials of 10 males (for a collection of 3-day-old flies for RNAseq). The aging cohort was maintained in vials, flies were tipped to new vials every 2–3 days without anesthesia, and periodically the entire cohort was anesthetized and rearrayed into groups of 40 animals in vials. Dead animals were counted daily. Supplementary Table 1 provides further description of how the aging cohort was treated. We elected to employ males for our experiment for practical reasons; flies can be transferred to fresh vials less often since media is not degraded by egg laying and larval development.

Flies were raised and maintained in standard narrow fly vials (Fisher Scientific, AS515) containing ∼10 mL of a cornmeal-yeast-molasses media (see Supplementary Text 1) and kept in an incubator under the following environmental conditions: 25°C, 50% relative humidity, and a 12:12 light:dark cycle.

### Fly/tissue sampling

Multiple groups of flies were sampled from the population through the aging process, with animals for the first—day 3—timepoint coming from those vials initially holding 10 flies (see above). Flies were sampled without anesthesia via manual aspiration. Sampled flies were always given at least 24 h between CO_2_ anesthesia and sampling, and sampling always occurred 3–4.5 h after lights on. See Supplementary Table 1 for details on when flies were sampled. We note that since flies were initially collected over a 2-day window (see above), on day 3 of the experiment, flies are actually 1–3 days old (on day 17, flies are 15–17 days old and so on). To simplify presentation below, we refer to sampling points solely as the day of the experiment (which is equivalent to the maximum age of the flies sampled that day).

On collection, groups of 10 male flies were moved into screw-top tubes, flash frozen in liquid nitrogen, and kept at −80°C. Subsequently—after the entire cohort had died and flies from all timepoints had been sampled—tubes were removed from the freezer into aluminum dry bath blocks held on dry ice. We then went through the tubes one by one, subjected each to liquid nitrogen, briefly vortexed to separate heads/bodies, poured the body parts into the flat surface of an aluminum dry bath block placed on dry ice, and used a paint brush to manually collect heads into a fresh screw-top tube. These destination tubes were prefilled with 4–6 glass beads (BioSpec Products, 11079127) that had been previously washed in bleach and thoroughly rinsed with distilled water. We elected to focus on head tissue since heads can be removed easily in *Drosophila* without dissection, and heads are enriched for neurological cells, which are of particular interest in aging.

### RNA isolation, sequencing library preparation, and sequencing

RNA was isolated using the Zymo Direct-zol MicroPrep kit (Zymo, R2062), largely following the manufacturer's protocol (see Supplementary Text 2). Samples were isolated over 6 batches, replicate samples from a given timepoint were isolated in different batches, and RNA quantity was measured using a NanoDrop ND-1000.

Forty-eight RNA samples were used to generate poly-A selected mRNA sequencing libraries; 14 timepoints had 3 replicates each, while 1 (day 59, the final timepoint) had 6 replicates. We used 200–500 ng of total RNA from these samples to initiate half-reaction volume mRNA sequencing library construction (Illumina TruSeq stranded HT kit using dual indexing), generating libraries across 2 batches of 24 samples each. Libraries were quantified using a Qubit fluorometer, and a subset of 8 libraries from each batch was run on an Agilent TapeStation. Each of these libraries showed a single library peak, no evidence of adapter dimers, and estimated average fragment sizes of 277–294 bp. See Supplementary Table 2 for details on RNA isolation/library preparation batching and quantification. Given the relatively uniform fragment sizes, equal quantities of all 48 libraries were pooled together. The final 48-plex pool had an average fragment size of 289 bp and was run over 2 Illumina NextSeq550 PE75 flow cells, yielding a total of over 635 million read pairs, with an average of 13.2 million per sample (range = 9.0–17.4 million).

### Quantifying expression level and identifying expression changes during aging

Reads from each of the 48 samples were processed using Salmon (Patro *et al*. 2017), employing release BDGP6.32 of the *D. melanogaster* transcriptome/annotation from Ensembl. Salmon quantifications were then summarized to gene level using R/tximeta (Love *et al*. 2020). To identify genes whose expression significantly changed with aging (adjusted *P*-value < 0.05), we used R/DESeq2 (Love *et al*. 2014), executing 3 different analyses, in each case associating expression with a different variable. First, we identified genes associated with chronological age (the “Day analysis”) by treating the age of the flies in each RNAseq sample as a continuous variable. Second, we identified genes associated with physiological age (the “Survival analysis”) by using the fraction of dead animals in the entire cohort at the point flies were sampled for RNAseq as a continuous variable (Supplementary Table 3). Third, we identified genes showing expression variation through aging by considering the 15 sampling points as levels of a categorical variable (the “Sampling Point analysis”). This final analysis sought to identify genes with expression patterns not easily captured by the 2 continuous variables (Day and Survival).

### Clustering genes by their expression trajectories

Our 3 differential expression analyses (see above) collectively identified 6,142 unique genes, and we sought to cluster these genes into groups based on their expression trajectories through aging. To focus on expression trajectories and avoid confounding with varying expression levels, we standardized the expression counts of each gene via *Z*-scores. Briefly, for each gene, we calculated the mean expression across replicates for each sampling point, along with the overall mean expression across all sampling points and replicates. We then subtracted the overall mean from the mean of each sampling point and divided by the overall standard deviation.

The calculated *Z*-scores for the 6,142 genes were used to create a dissimilarity matrix using the Pearson correlation method in R/factoextra (Kassambara and Mundt 2020). We used this dissimilarity matrix for hierarchical clustering of our identified genes and then “cut” the resulting dendrogram into a designated number of gene groups/clusters. This was done using the hclust and cutree functions from the R/stats package (R Core Team 2021).

There are a variety of ways to cut a gene dissimilarity matrix into clusters, and variation in the approach and parameters will yield different numbers and sizes of clusters. Our goal was to examine whether clusters of genes with similar expression trajectories were enriched for particular properties [e.g. gene ontology (GO) terms]. To facilitate this, we sought to avoid clusters with either very small or very large numbers of genes, so targeted clusters with between 50 and 500 genes. After exploring several methods, we grouped our 6,142 differentially expressed genes into 28 clusters (see Supplementary Table 4 and Fig. 2 for more information).

### Summarizing and classifying cluster expression trajectories

For each of the 28 clusters, we created a representative expression curve by smoothing the mean *Z*-score from all genes in the cluster for each sampling point (Fig. 4). The smoothing was executed using geom_smooth from R/ggplot2 (Wickham 2016) and spline modeling with rcs from R/rms (Harrell 2023). The resulting 28 curves show a diversity of trajectories, with some being generally linear, while others show a more complex pattern. To classify the cluster trajectories, we ran a linear regression between the mean *Z*-scores and the age of the sampled flies. A trajectory was designated as “Linear” if the *P*-value was <0.002 (0.05/28) or “Complex” otherwise. (Repeating this analysis using survival, or the numbered sampling points, SP1–SP15, yields the same designations.) Linear trajectories were further designated as “Up” (gene expression increases with aging) or “Down” (gene expression decreases with aging) based on the sign of the linear regression coefficient. To simplify subsequent discussion, clusters are named with these classifications (i.e. Complex, LinearUp, or LinearDown) and given with numeric codes based on the cluster position within the dendrogram (bottom to top in Fig. 3 and left to right in Supplementary Fig. 3). See Supplementary Table 5 for details on the trajectory-designating linear regression analyses.

### Cluster-specific enrichment analyses

To understand whether genes with similar expression trajectories share similar properties/functions, we used PANGEA (Version 1.1 beta December 2022) (Hu *et al*. 2023), an online gene set enrichment tool that can perform GO analysis, identify enrichment of particular gene groups or pathways, and—importantly for our needs—can execute analyses on multiple gene lists simultaneously. For each cluster, we ran 6 separate enrichment analyses, examining 3 *Drosophila* GO subsets (SLIM2 GO BP—biological process, SLIM2 GO CC—cellular component, and SLIM2 GO MF—molecular function), 2 collections of gene groups (DRSC GLAD and FlyBase), and the REACTOME pathway set. For each analysis, we used a custom background set of 13,303 genes that included only those with at least one mapped read in our dataset. We identified terms significantly enriched in each cluster using a Benjamini–Hochberg false discovery rate of 0.05. Each of the PANGEA tables is available as Supplementary Tables 6–11, and our enrichment analysis code is available at https://github.com/Hanson19/RNAseq-Aging.

### Comparing trajectory-based, multitimepoint analysis results to analyze contrasting groups of young and old animals

Our design differs from some previous examinations of age-related expression in that we generated expression data from many points through the aging process. To examine what might be gained from our approach, we reanalyzed our data after dropping the bulk of the timepoints. The samples from day 3 and day 6 (*n* = 6) collectively made up our “young” sample, while our “old” sample came from the last collection day, day 59 (*n* = 6). Using R/DESeq2 (Love *et al*. 2014), we identified genes whose expression changed significantly between these age groups and determined if gene expression increased or decreased over time. We subsequently repeated this analysis, comparing the days 3 + 6 “young” timepoint against every sequential pair of older timepoints (e.g. we compared days 3 + 6 with days 10 + 14, days 3 + 6 with days 14 + 17, and so on).

### Comparison with previous *Drosophila* aging genome-wide expression studies

We compared our set of 6,142 multitimepoint significant genes with those identified in 8 previously published 2-timepoint aging expression studies (Supplementary Table 13). These papers vary in the strains/populations employed, the sex of the animals targeted, the tissue that was employed, and the actual timepoints during aging that were sampled. We validated all gene IDs using FlyBase (FB2024_01) (Jenkins *et al*. 2022), identified genes shared between our study and these previous works, and compared the expression change reported in the previous studies (up or down in expression with age) with the expression trajectories that these genes were grouped into in our study (LinearUp, LinearDown, and Complex; Supplementary Fig. 11).

### Shiny apps to enable data exploration

Our analyses generated a considerable amount of data, and to make our results more accessible, we developed 2 interactive apps using R/shiny (version 1.8.0) (Chang *et al.* 2023). The Gene and Cluster app allows users to look up specific genes, receive information about whether the gene was identified in our analysis, and if so in which cluster it was found, and what its expression trajectory is over time. The Cluster Enrichment app allows users to select specific clusters, or sets of clusters, and identify any enriched terms. For more information on how to run and use these apps, see Supplementary Text 3 and 4.

## Results and discussion

### Over 6,000 genes show expression change during aging

**Fig. 1.**
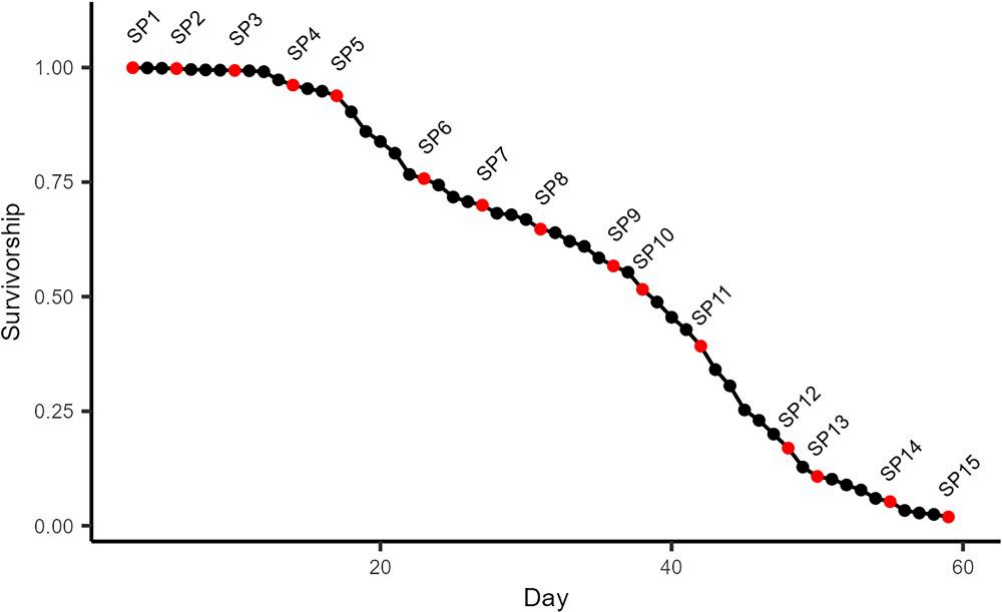
Kaplan–Meier survivorship curve for over 5,000 males from the inbred A4 strain. Each point represents 1 day of the experiment, with black points denoting days when only the number of dead flies were counted, and red points denoting those days when flies were also sampled for subsequent RNA isolation (SP1–SP15).

We aged a cohort of 5,330 *D. melanogaster* males from a single inbred strain, recorded the number of fly deaths each day, and collected several replicates of 10 flies at 15 timepoints throughout the adult lifespan. Samples were collected every ∼4 days between day 3 (99.9% flies alive) and day 59 (1.92% alive) to roughly evenly sample flies throughout lifespan (see Kaplan–Meier survivorship curve in Fig. 1 and details on the timing of the sampling in Supplementary Table 3). RNA was isolated from the heads of collected flies, converted into RNAseq libraries and sequenced, and subsequently reads were assembled to the transcriptome to quantify gene expression.

Three separate analyses were used to identify genes with significant expression changes through aging. For each of 2 analyses, we associate gene expression with a different continuous variable, either the chronological age of the flies on the day samples were collected (Day analysis) or the survivorship of the aging cohort upon sampling (Survival analysis). In the 3rd analysis, we associated expression with a single categorical variable with 15 levels (SP1–SP15) that reflect the sampling points when flies were collected (Sampling Point analysis). This 3rd analysis has the potential to identify genes missed by the pair of continuous variable analyses, since expression variation of some genes may not follow a simple, continuous temporal pattern.

**Fig. 2.**
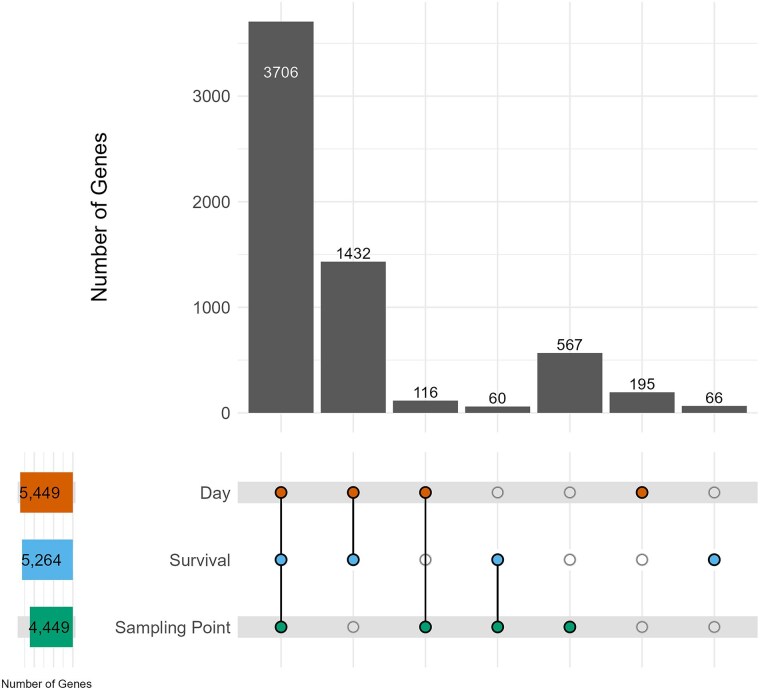
Identification of 6,142 genes with age-related gene expression changes. We identified genes whose expression was significantly associated with Day of life (*n* = 5,449), with Survival (*n* = 5,264), and with Sampling Point (*n* = 4,449). The upper bar chart shows the number of significant genes identified, with colored circles below showing which analysis the genes were identified in (3,706 genes were identified in all 3 analyses, 1,432 genes were identified in both the Day and Survival analyses, and so on).

The 3 analyses collectively identified 6,142 unique genes that were differentially expressed through lifespan (Fig. 2), representing a little under half of the genes with detectable expression in our study (*n* = 13,303). The analyses individually identified 5,449 (Day), 5,264 (Survival), and 4,449 (Sampling Point) genes. Around 60% of the genes (3,706) were identified by all 3 analyses, and an additional 28% (1,432) were identified by both continuous variable analyses, Day and Survival. The large overlap of genes identified in both the Day and Survival analyses—well over 90% of the genes identified in each analysis are shared among the 2—is expected since age in days and survivorship are strongly correlated (*r* = 0.98, *P* < 10^−9^; Supplementary Fig. 1). The Sampling Point analysis identified the most genes unique to one analysis—567 (9% of the total number of unique genes identified)—demonstrating its utility in capturing genes not easily found by either continuous variable analysis.

### A diversity of gene expression trajectories through aging

**Fig. 3.**
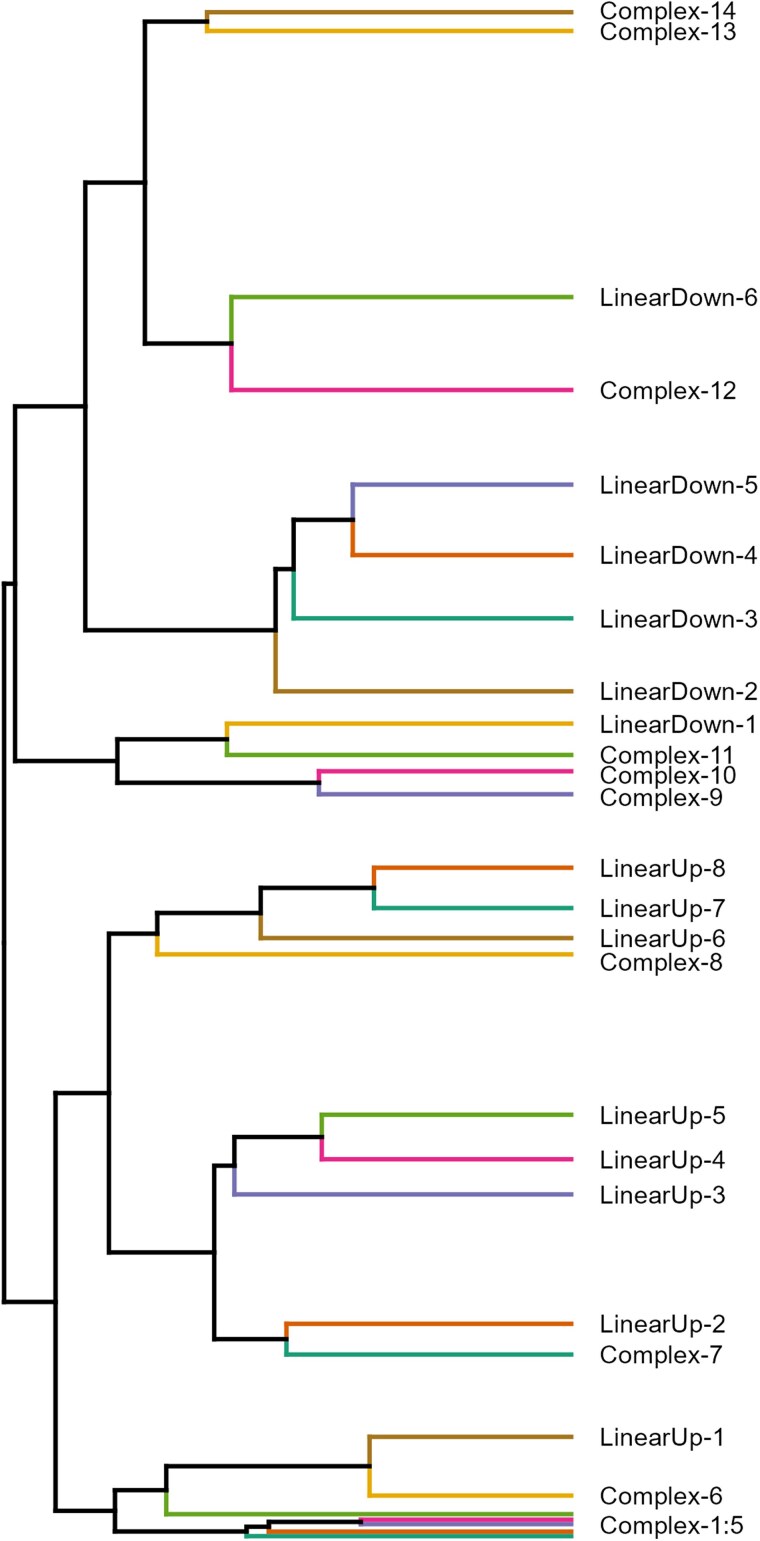
Clustering all 6,142 age-related genes into 28 clusters via their expression trajectories through lifespan. A simplified dendrogram representing the hierarchical clustering of our gene expression trajectory data. Each horizontal colored line represents all those genes in each of our 28 clusters; the closer clusters are to each other on the plot, the more similar their expression trajectories (see Fig. 4). Each cluster is named based on their expression trajectory (LinearUp, LinearDown, and Complex). Complex-1 to Complex-5 clusters are not individually labeled since they are very close together in the plot. Supplementary Fig. 3 shows the full dendrogram highlighting the relationships among all 6,142 genes.

**Fig. 4.**
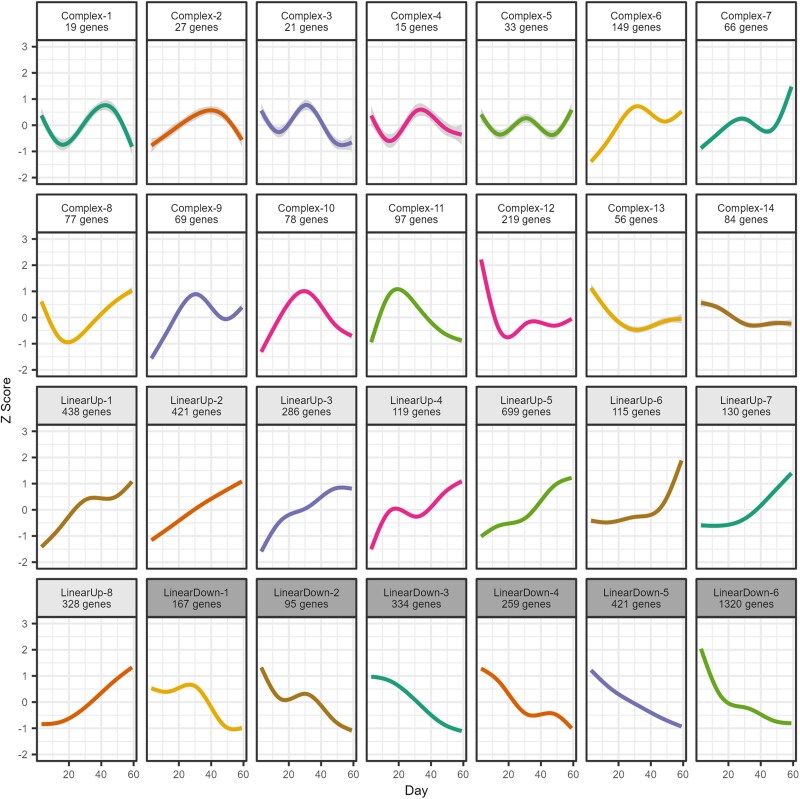
Representative expression trajectories for all 28 clusters. Within a cluster, we calculated the average *Z*-score (*y*-axis) over genes for each timepoint and present a smoothed curve through those points highlighting the cluster-specific changes in gene expression over time in days (*x*-axis). We determined whether each cluster-specific set of mean *Z*-scores was statistically associated with age and used this information to designate each cluster as LinearUp or LinearDown (a significant association and expression either increases or decreases over time) or as Complex (there was no significant association between expression and age). This led to 14 Complex, 8 LinearUp, and 6 LinearDown clusters.

Genes with similar functions, or that function in the same pathways, might be expected to share similar expression trends over time (e.g. Eisen *et al*. 1998; Zhang *et al*. 2004). To enable investigation of this, we normalized the expression trajectories of all 6,142 identified genes via *Z*-scores and clustered them into 28 groups (Fig. 3; Supplementary Fig. 3). Based on the results of linear regressions of cluster-specific expression patterns (Fig. 4) against time in Days, each cluster was assigned a trajectory designation of LinearUp or LinearDown (the mean cluster expression is significantly associated with time and either goes up or down over time, respectively) or Complex (there is no significant association with time after correcting for multiple testing). See the *Materials and methods* section for further details on this process and Supplementary Table 5 for statistical information. Over 80% of genes fall into the 6 LinearDown (2,596) and 8 LinearUp (2,536) clusters, with the remaining 1,010 genes split among 14 Complex clusters (Fig. 4). In general, the Complex clusters harbor considerably fewer genes than the more linear clusters.

Clearly, the LinearDown and LinearUp clusters do not show perfect linear patterns of expression; for instance, genes in LinearDown-1 (Fig. 4) show a slight increase in expression in midlife, before decreasing in expression toward late life. However, the curves for the linear clusters typically appear more linear than those of the Complex clusters (compare LinearUp-8 with Complex-1) and primarily show monotonic increases/decreases in expression over time (see LinearUp-7). Furthermore, among the Complex class of clusters, we see a great deal of variation in expression trajectory; many are decidedly nonlinear and show wave-like patterns (e.g. Complex-5) or are curved with the highest expression in midlife (e.g. Complex-10). However, some Complex cluster are somewhat linear and exhibit patterns not dissimilar to those of LinearUp/Down clusters (e.g. compare Complex-6 with LinearUp-4). We recognize that our “linear” clusters are not perfectly linear and do vary in their trajectories over time and that our “complex” clusters exhibit a wide spectrum of trajectories. However, to simplify presentation, we elected to employ a straightforward trajectory-based naming scheme for the clusters we identify (i.e. LinearUp, LinearDown, and Complex).

### Genes with complex expression trajectories are often identified via the sampling point analysis

We examined the relationship between the statistical analyses a gene was identified in (Day, Survival, and Sampling Point), the cluster in which it resides, and the trajectory it was assigned (LinearUp, LinearDown, and Complex). More than 75% of the genes identified solely in the continuous variable analyses (i.e. Day only, Survival only, or both) reside in the LinearUp/Down clusters, whereas ∼43% of those genes found in Complex clusters were uniquely identified in the Sampling Point analysis (Supplementary Fig. 4). This result does not appear to be driven by specific clusters (Supplementary Fig. 5). This again implies that the Sampling Point analysis has the potential to identify genes whose age-related changes in expression are difficult to capture with linear analyses based on chronological time or survivorship.

### Identification of known aging-relevant genes

Our analyses identified many genes previously associated with aging in *Drosophila*. We identified 107/176 genes associated with the GO term “determination of adult lifespan” (GO:0008340), with at least one such gene being present in 24 of the 28 clusters. For instance, we identified *I’m not dead yet* (Complex-6, FBgn0036816), a transporter of Krebs cycle intermediates, which when mutated increases lifespan via a mechanism resembling the effect of caloric restriction (Rogina *et al*. 2000). We found insulin signaling genes, including *chico* (LinearUp-8, FBgn0024248) and *Insulin-like peptide 2* (Complex-11, FBgn0036046), for which loss-of-function mutations increase lifespan (Clancy *et al*. 2001; Grönke *et al*. 2010). We also identified the heat shock proteins *Hsp22* (LinearUp-6, FBgn0001223), *Hsp26* (LinearUp-8, FBgn0001225), and *Hsp68* (LinearUp-5, FBgn0001230), which when overexpressed can increase lifespan (Wang *et al*. 2003, 2004; Morrow *et al*. 2004).

### Exploring the biological functions of expression trajectory clusters

To understand if genes with similar expression trajectories share similar functions, we executed a series of enrichment analyses using the software PANGEA (Hu *et al*. 2023), identifying enriched GO terms, gene groups, and pathways within each of our 28 clusters. GO terms are derived from the *Drosophila* GO subsets available within PANGEA (terms are indicated below by the “GO” stem), the gene groups are from DRSC GLAD (“GLAD”) and FlyBase (“FBgg”), and pathways come from Reactome information (“R-DME”). In total, we identified 732 unique enriched terms, 595 of which are specific to just one of our expression trajectory designations (i.e. LinearUp, LinearDown, and Complex), suggesting our 3 designations represent largely distinct sets of biological processes. Furthermore, most of the unique enrichment terms are found in just a single cluster, with only 67 terms being shared by multiple clusters. (The PANGEA output tables are available in Supplementary Tables 6–11.)

#### Confirming common aging-related gene expression patterns

In *Drosophila* aging expression studies, it is commonly observed that both general stress response genes and immunity genes increase in expression with age (Pletcher *et al*. 2002; Landis *et al*. 2004; Girardot *et al*. 2006; Carlson *et al*. 2015; Highfill *et al*. 2017; Bordet *et al*. 2021; Zane *et al*. 2023), and we recapitulate this finding. Five of our clusters are enriched for genes that respond to stress (GO:0006950), all of which are designated LinearUp (LinearUp-2, 3, 5, 7, and 8; Fig. 4). Previous studies have seen that heat shock proteins increase in expression with age (King and Tower 1999; Landis *et al*. 2004; Manière *et al*. 2014), and we see these genes (FBgg0000501) are enriched in 2 LinearUp clusters (LinearUp-5 and 7). Of the 5 clusters that are enriched for stress response genes, 3 are more specifically enriched for immune response genes (GO: 0006955; LinearUp-2, 5, and 8). LinearUp-5 and 8 are enriched for antimicrobial peptides (FBgg0001101), including *Attacin-A* (LinearUp-8, FBgn0012042), *Listericin* (LinearUp-8, FBgn0033593), and *Drosocin* (LinearUp-5, FBgn0013088), which are commonly found to increase in expression with age in *Drosophila* expression studies (Landis *et al*. 2004; Lai *et al*. 2007; Carnes *et al*. 2015; Highfill *et al*. 2017; Bajgiran *et al*. 2021; Bordet *et al*. 2021; Zane *et al*. 2023).

A decrease in cognitive ability with advanced age has been reported in *D. melanogaster* (Tamura *et al*. 2003; Haddadi *et al*. 2014; Pacifico *et al*. 2018), mice (Kubanis *et al*. 1981; Lamberty and Gower 1990; Healy *et al*. 2024), and rats (Rowe *et al*. 1998; Sagheddu *et al*. 2024). Similarly, age-related neurodegeneration is commonly observed, with decreased neurogenesis with aging being reported in both mice (Maslov *et al*. 2004) and rats (Kuhn *et al*. 1996), and synaptic deterioration is seen in the motor neurons of aged *C. elegans* (Liu *et al*. 2013). Three of our clusters—LinearDown-3, 4, and 6—are enriched for genes that are found in synapses (GO:0045202) and are involved in synapse organization (GO:0050808), as well as those associated with cognition (GO:0050890) and nervous system development (GO:0007399). LinearDown-4 and 6 are also enriched for genes encoding voltage-gated potassium and sodium channel subunits (FBgg0000506, FBgg0000595). Notably, LinearDown-6 harbors *Atpα* (FBgn0002921), a gene encoding a subunit of the NA^+^/K^+^ exchanging ATPase pump, and which has been shown to affect lifespan (Palladino *et al*. 2003).

A decrease in the expression of genes involved with the ETC is a common finding in samples of aged individuals (Pletcher *et al*. 2002; Landis *et al*. 2004; Girardot *et al*. 2006; Highfill *et al*. 2017; Bordet *et al*. 2021; Zane *et al*. 2023). Such a decrease could lead to reduced ATP production, disruption of the NAD+/NADH ratio, and cellular senescence (Lenaz *et al*. 1997; Miwa *et al*. 2014; Miwa *et al*. 2022). Our study supports a decrease in ETC-related gene expression; genes in Complex-12 and LinearDown-6 show an overall decrease in expression across lifespan (Fig. 4), and these clusters are adjacent to each other in the dendrogram resulting from hierarchical clustering (Fig. 3; Supplementary Fig. 3). These 2 clusters are enriched for genes encoding mitochondrial ETC complexes I and V (FBgg001836, FBgg0001849). LinearDown-6 is also enriched for genes that are part of mitochondrial complexes III and IV (FBgg0001850, FBgg0001847). A series of ETC-related genes shown to influence lifespan are also present in these clusters; these include *ND-20* (Complex-12, FBgn0030718) and *ND-SGDH* (LinearDown-6, FBgn0011455) (Copeland *et al*. 2009), *levy* (LinearDown-6, FBgn0034877) (Liu *et al*. 2007), and *ATPsynD* (LinearDown-6, FBgn0016120) (Sun *et al*. 2014). Additionally, LinearDown-6 harbors *stress-sensitive B* (FBgn0003360), which is involved in the transport of ADP and ATP in and out of the mitochondrial matrix and when mutated shortens lifespan (Celotto *et al*. 2006; Reynolds 2018).

#### A distinction between cytosolic and mitochondrial ribosomal gene expression responses

A decrease in the expression of ribosomal proteins and ribosomal biogenesis genes with age has been documented in a number of yeast studies (Yiu *et al*. 2008; Philipp *et al*. 2013; Kamei *et al*. 2014; Janssens *et al*. 2015; Choi *et al*. 2018), with 3 of these showing a reduction in cytosolic ribosome (GO: 0022626) gene expression over time (Yiu *et al*. 2008; Philipp *et al*. 2013; Choi *et al*. 2018). One fly study we identified (Doroszuk *et al*. 2012) observed a similar response, with enrichment of ribosome-related ontology terms in genes showing a reduction in expression with age in a given strain, including genes involved in ribosome biogenesis (GO:0042254) and mitochondrial ribosome (GO:0005761).

In the analyses of our dataset, we saw enrichment of ribosomal-related ontology terms in 6 clusters. Five of these clusters show a general increase in gene expression with age (LinearUp-1, 2, 4, 6, and Complex-6; see Fig. 4) and include genes associated with the terms ribosome biogenesis (GO:0042254), rRNA processing (R-DME-72312), cytoplasmic ribosomal proteins (FBgg0000141), and structural constituent of ribosomes (GO:0003735). The 6th (LinearDown-6) shows reduced expression with age and shows enrichment of mitochondrial ribosomal proteins (FBgg0000059). Thus, in an apparent contrast with prior results, it appears that in our data many ribosome-associated genes increase in expression with age, while only mitochondrial ribosomal protein genes decrease with advanced age.

**Fig. 5.**
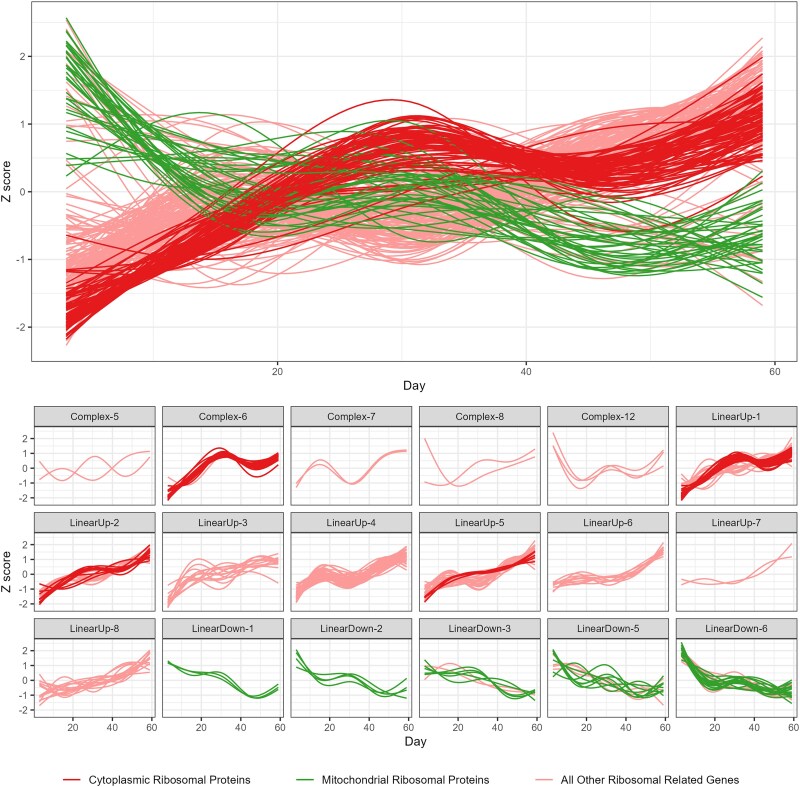
Expression trajectories of ribosome-related protein-coding genes. We examined the expression patterns of mitochondrial ribosomal protein genes (*n* = 36, green), cytoplasmic ribosomal protein genes (*n* = 80, red), and all other ribosomal-related genes (*n* = 154, pink). All mitochondrial ribosomal genes are within LinearDown clusters, all cytoplasmic ribosomal genes are found within LinearUp cluster or Complex-6 (which has an overall increase in expression), and 140/154 of the remaining genes are found within LinearUp or Complex clusters.

To examine this further, we extracted from our complete set of 6,142 differentially expressed genes all those affiliated with any ribosome-related term (*n* = 270, see Supplementary Table 12 for list of terms). We plotted the age-related expression of these 270 protein-coding genes, separating out mitochondrial ribosomal proteins (*n* = 36, FBgg0000059), and can clearly see that while all mitochondrial ribosomal protein genes go down in expression with age, nearly all other ribosomal genes (220/234) increase in expression with age (Fig. 5). Furthermore, just contrasting cytoplasmic (*n* = 80, FBgg0000141) and mitochondrial (*n* = 36, FBgg0000059) ribosomal proteins, we see the former all go up, and the latter all go down in expression with age (Fig. 5).

**Fig. 6.**
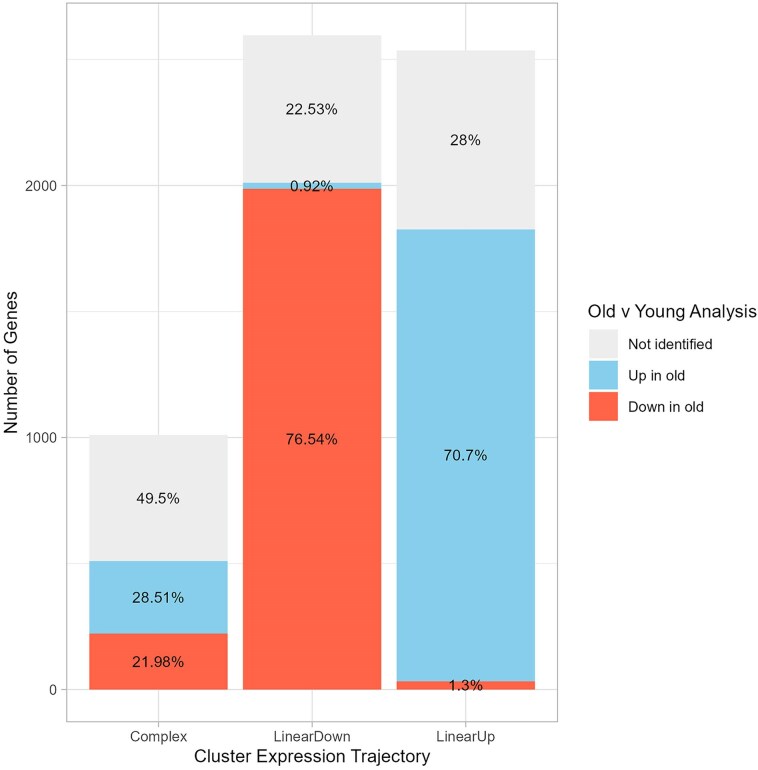
Differences between a multitimepoint and a 2-timepoint analysis. Our 3 trajectory-based analyses revealed >6,000 differentially expressed genes that were grouped into 3 trajectories (Complex, LinearDown, and LinearUp). We compared these results with a reanalysis of a subset of the same data, where we directly contrasted expression between young (days 3 + 6) and old (day 59) samples. Each vertical bar depicts the fraction (in the figure) and the number (*y*-axis) of genes in each of our expression trajectories that are absent in the young vs old test (gray), are significantly upregulated in old animals (blue), or are significantly downregulated in old animals (red). Most genes with linear trajectories are reidentified, and ∼99% of those show the expected direction of change with age. However, only ∼50% of the Complex trajectory genes are reidentified in the 2-timepoint analysis, and these are split between those that appear to increase or to decrease in expression with age.

A *D. melanogaster* brain-specific, single-cell RNAseq study (Davie *et al*. 2018) appears to support our finding that—outside of mitochondrial ribosomes—ribosomal genes increase in expression with age. First, Davie *et al*. report that the cytosolic small ribosomal subunit (GO: 0022627) gene *stubarista* (FBgn0003517) is statistically upregulated during aging; we also identified this gene in our LinearUp-5 cluster. Second, while Davie *et al*. see a reduction in the overall levels of RNA and transcription through aging—a result seen previously (Tahoe *et al*. 2004) and which we also observed (Supplementary Table 2 and Fig. 6)—on average, ribosomal protein genes show a lower decline in expression than other genes (see Fig. 5c in Davie *et al*. 2018). In a bulk RNAseq analysis framework, this result would be expected to translate to a relative increase in the expression of ribosomal proteins over time. Nonetheless, that we see a result that contrasts with some of the prior work on gene expression changes through aging is intriguing and worthy of future examination.

#### Various metabolic processes are enriched in some complex clusters

Many of our Complex clusters have small gene counts and relatively few enriched terms. However, 8/14 Complex clusters (2, 3, 4, 6, 7, 9, 10, and 12) are enriched for genes associated with metabolism (R-DME-1430728 and GLAD:24593). While this term is broad, when we focus on individual clusters more specific metabolic functions are evident. As described earlier, Complex-12 is enriched for genes involved with the ETC. Complex-2, 6, and 10 are specifically enriched for genes involved in the pentose phosphate pathway (R-DME-71336), a glucose catabolism pathway that produces NADPH and ribose sugars for nucleotide synthesis (Stincone *et al*. 2015). Notably, Complex-2 harbors *Pgd* (FBgn0004654) and *G6pd* (FBgn0004057), which are the 2 reducing enzymes involved in the pentose phosphate pathway (Geer *et al*. 1974; Gvozdev *et al*. 1976). Complex-3 is uniquely enriched for genes involved in galactose catabolism (R-DME-70370) and glycogen synthesis (R-DME-3322077), one of which—*Agbe* (FBgn0053138)—is involved in lifespan (Paik *et al*. 2012). That all of these metabolic pathways/genes emerged from our Complex clusters suggests that nonlinear changes in metabolic activity occur throughout lifespan, as has been suggested in a large, multiomic study in humans (Shen *et al*. 2024).

#### Enrichment for protein folding, modification, and transport in the LinearUp-7 cluster

LinearUp-7—which shows only limited change in expression for the first third of life, followed by increasing expression until end of life—is enriched for multiple terms involved with protein folding and modification and transporting proteins from the endoplasmic reticulum to the golgi apparatus. We see enrichment for chaperones and cochaperones (FBgg0001643), and heatshock proteins (see above), which can bind onto unfolded proteins (GO:0051082) and help correctly fold them (GO:0006457). Additionally, LinearUp-7 is enriched for genes involved in posttranslation protein modification pathways (R-DME-597592). LinearUp-7 is the only cluster to be enriched for genes associated with both the endoplasmic reticulum (GO:0005783) and the golgi apparatus (GO:0005794). The cluster also includes genes that are involved in transport between these 2 organelles and is enriched for both coat protein complex I (FBgg0000087) and II (FBgg0000116) genes and genes involved in ER-to-Golgi anterograde transport (R-DME-199977) and Golgi-to-ER retrograde transport (R-DME-8856688). These enrichment patterns further demonstrate that genes with similar roles can have very similar temporal expression patterns.

### Multitimepoint trajectory-based datasets offer more detail than 2-timepoint studies

Often aging expression studies compare “young” and “old” samples, and we sought to reanalyze our data in this framework to discover what is gained from a multitimepoint approach. Our “young” timepoint combined all the day 3 and day 6 samples (to achieve a sample size of 6), and our “old” timepoint used the six day 59 samples. Contrasting these 2 sets of samples yielded 4,533 differentially expressed genes. Of the 6,142 genes identified in our multitimepoint analysis, 4,347 (∼71%) were reidentified in this 2-timepoint analysis. The reduction in the number of genes is likely a combination of the switch in analytical approach and a simple loss of power (since we have gone from 48 to 12 samples). Considering the assigned expression trajectories from our multitimepoint analysis, the 2-timepoint analysis recovered 77.5% of the LinearDown cluster genes and 72% of the LinearUp genes, but only 50.5% of the Complex genes (Fig. 6). As anticipated, genes that do not exhibit a straightforward, monotonic increase/decrease in expression through lifespan are much less likely to be identified when sampling is restricted to very young and very old animals.

A challenge with a 2-timepoint analysis is that the only conclusion one can draw about the expression change is that it goes up or down with age. For those genes present in LinearUp/LinearDown clusters that were replicated in the 2-timepoint analysis, the inferred direction of the expression change matched expectations 98–99% of the time (Fig. 6), and this trend is consistent across the different LinearUp/LinearDown clusters (90–100% matched) (Supplementary Fig. 7). However, for the 50.5% of Complex genes that were reidentified in the 2-timepoint analysis, 56% of them showed an increase in expression in old samples, and 44% showed a decrease (Fig. 6). Examining the 2-timepoint expression change direction calls across Complex clusters reveals cluster-to-cluster variation (see Supplementary Fig. 7), and in most cases, reidentified genes within a given Complex cluster exhibit the same direction of expression change in the 2-timepoint analysis (see Supplementary Fig. 7). This likely reflects the cluster-specific nature of each Complex expression trajectory and the precise expression levels at the start and end of our experiment. For instance, Complex-7 shows a general increase in expression over time (Fig. 4), and all genes reidentified in the 2-timepoint analysis are marked as increasing in expression (Supplementary Fig. 7).

In our initial analysis we chose days 3 + 6 and day 59 to represent our “young” and “old” samples. However, studies have used flies of quite different ages to represent young and old animals (Landis *et al*. 2004; Girardot *et al*. 2006; Carnes *et al*. 2015; Bajgiran *et al*. 2021; Bordet *et al*. 2021). To understand the impact of varying the “old” timepoint, we repeated the 2-timepoint analysis several times. In each case, we fixed the young timepoint using our days 3 + 6 data and derived the old timepoint from 2 sequential sampling days (e.g. days 10 + 14, days 14 + 17, days 17 + 23, and so on) such that each analysis compared 2 sets of 6 samples. We then compared the differentially expressed genes that emerged from these analyses, along with the inferred expression change, with our initial days 3 + 6 vs day 59 analysis. As might be expected, using old sampling points that occur earlier in life results in fewer significant differentially expressed genes (Supplementary Fig. 8); there has simply been less time for change. As the old timepoint moves later in life, there is increasingly greater overlap with the days 3 + 6 vs day 59 analysis, and 97–99% of the shared genes show the same change in expression (Supplementary Fig. 8). Nonetheless, every alternative analysis with a different old timepoint identified genes we initially identified in our multitimepoint trajectory analysis, but that were missed in our initial 2-timepoint analysis (Supplementary Fig. 8). Particularly in those analyses using old timepoints that occur earlier in life (up to day 36, a little over halfway through the lifespan of our cohort), such genes are often associated with our multitimepoint Complex trajectory clusters (Supplementary Fig. 9). Between 9% and 17% of Complex genes found using the earlier alternative old timepoints were not found in our initial days 3 + 6 vs day 59 test. It is likely that the observed change in the expression of these genes over time is highly dependent on the exact timepoints chosen. In general, our analyses clearly demonstrate that the age when individuals are sampled impacts the genes identified.

### Comparison with other aging expression studies in flies

We compared the outcome of our multitimepoint analysis with 9 datasets from 8 previously published expression analyses in *D. melanogaster* (Landis *et al*. 2004; Girardot *et al*. 2006; Lai *et al*. 2007; Carnes *et al*. 2015; Highfill *et al*. 2016; Bajgiran *et al*. 2021; Bordet *et al*. 2021; Zane *et al*. 2023). These were all 2-timepoint studies that varied in the sex of the flies, the target tissue, and the sampling points employed (Supplementary Table 13). Around 95% of the genes resulting from our multitimepoint analysis were identified in at least one of these datasets, and the number of previous studies that identified a given gene was not clearly associated with the trajectory designation (LinearUp, LinearDown, and Complex) we assigned (Supplementary Fig. 10). The high rate of gene reidentification across studies is notable given the diversity of the study designs in terms of fly sex, the tissue targeted, the sampling points used (Supplementary Table 13), and the likely many differences in the precise rearing/maintenance conditions employed. That the same sets of genes are regularly identified suggests some similarity across genotype/sex/tissue in the age-related expression profile (Izgi *et al*. 2022).

Similar to the comparisons between multitimepoint and 2-timepoint analyses within our own dataset (above), when we look at genes in our LinearUp/LinearDown clusters that were identified in prior studies, they broadly show the direction of expression change we would predict (Supplementary Fig. 11). However, as might also be expected, the fraction of such genes showing the predicted expression change in a prior study is often lower than it is in our within-study methodological comparison (compare Fig. 6 with Supplementary Fig. 11). The highest fraction of LinearUp/LinearDown genes showing the expected expression change in a prior study—95–98%—is with the study by Highfill *et al*. (2016; Supplementary Fig. 11), a study published by our group that used the same tissue type, employed a very similar fly maintenance environment, but targeted females rather than males. The other prior studies we examined varied more in their design, and these differences likely contribute to shared genes more often showing mis-matched expression changes (see Supplementary Fig. 11).

### Benefits of a multitimepoint, trajectory-based transcriptomics approach to exploring dynamic biological processes

It is increasingly clear that a complete view of the gene regulatory changes underlying a range of dynamic processes—from the response to infection (Schlamp *et al*. 2021) to cellular differentiation (Strober *et al*. 2019) to aging (Pletcher *et al*. 2002; Gheorghe *et al*. 2014; Shen *et al*. 2024)—requires a timecourse experimental design, interrogating samples taken throughout the process of interest. Here, we showed that a 2-timepoint, young vs old analysis can successfully identify and correctly infer the direction of expression change of many genes that have largely linear expression trajectories through aging. But, we also showed that the set of genes identified depends on the pair of timepoints chosen. Furthermore, we showed that by limiting the sampling to just 2 timepoints, we would have failed to identify almost half of the genes with more complex, nonlinear expression trajectories and would not have captured the nature of the trajectories for genes that were identified. Even for those genes in the clusters we consider to be “linear,” there is variation in rate of expression change with time, which can only be captured with a multitimepoint approach (Lu *et al*. 2004; Gheorghe *et al*. 2014; Haustead *et al*. 2016; Schaum *et al*. 2020; Shen *et al*. 2024).

Another major benefit of being able to consider the trajectory of gene expression is that it enables the identification of genes with similar longitudinal expression patterns and allows assessment of whether genes with similar patterns have similar functional/molecular properties. Our enrichment analyses support the contention that genes with similar expression trajectories share similar functions. Due to the multiple points we sampled throughout the aging process, around 70% of all significant, enriched GO terms we identified were unique to a single cluster. This specificity allowed us to more precisely characterize the expression patterns of various sets of genes with related functional roles, rather than only—with a 2-timepoint framework—being able to state that particular groups of genes are upregulated or downregulated with age. Better understanding of the dynamic molecular changes underling aging will facilitate a deeper understanding of the cellular and physiological changes that occur as organisms age.

### Caveats

We recognize that the scope of our results may be somewhat limited due to the use of only males from one inbred strain and the use of a single tissue type. Additionally, while our target tissue—the fly head—is enriched for brain/neuronal cells, since it also includes a mixture of other cell types, we lack true tissue specificity. This said, we were able to demonstrate some consistency over studies in the genes and expression patterns identified, despite these studies varying in multiple ways, making our results a useful resource for future aging investigations.

### Accessible data exploration via interactive Shiny apps

Our expression, gene enrichment, and comparative analyses generated a significant amount of data. Above we have only discussed a subset of our observations. To make the results more accessible—in addition to sharing our raw data, summary data, and analytical code—we have developed 2 interactive R/shiny apps that enable individuals to explore our gene identification, clustering, and cluster enrichment results. The apps allow users to look up specific genes, examine their expression trajectory through time in our cohort of flies, the cluster they belong to, and to explore enriched terms within and across clusters. See the *Materials and Methods* section, along with Supplementary Text 3 and 4 for more information on the development of the apps and how to access and use them.

## Data Availability

The A4 strain is available on request from the corresponding author. Raw FASTQ sequencing data are available from the NCBI SRA under BioProject accession number PRJNA1194574. All summary data and results are presented in supplementary files (available at GSA FigShare: https://doi.org/10.25387/g3.28439153), and all analysis code is available via GitHub (https://github.com/Hanson19/RNAseq-Aging).
